# Radon Hazard in Central Italy: Comparison among Areas with Different Geogenic Radon Potential

**DOI:** 10.3390/ijerph19020666

**Published:** 2022-01-07

**Authors:** Francesca Giustini, Livio Ruggiero, Alessandra Sciarra, Stan Eugene Beaubien, Stefano Graziani, Gianfranco Galli, Luca Pizzino, Maria Chiara Tartarello, Carlo Lucchetti, Pietro Sirianni, Paola Tuccimei, Mario Voltaggio, Sabina Bigi, Giancarlo Ciotoli

**Affiliations:** 1 National Research Council, Institute of Environmental Geology and Geoengineering, CNR-IGAG, 00015 Rome, Italy; francesca.giustini@igag.cnr.it (F.G.); pietro.sirianni@igag.cnr.it (P.S.); voltaggiomario@gmail.com (M.V.); 2 Istituto Nazionale di Geofisica e Vulcanologia, 00143 Rome, Italy; livio.ruggiero@ingv.it (L.R.); alessandra.sciarra@ingv.it (A.S.); gianfranco.galli@ingv.it (G.G.); luca.pizzino@ingv.it (L.P.); 3 Dipartimento di Scienze della Terra, Sapienza-Università di Roma, DST-Sapienza, 00185 Rome, Italy; stanley.beaubien@uniroma1.it (S.E.B.); stefano.graziani@uniroma1.it (S.G.); chiaratartarello@hotmail.it (M.C.T.); carlo.lucchetti@hotmail.it (C.L.); sabina.bigi@uniroma1.it (S.B.); 4 Dipartimento di Scienze, Università di Roma Tre, 00154 Rome, Italy; paola.tuccimei@uniroma3.it

**Keywords:** soil gas and indoor radon, geogenic radon potential, risk assessment

## Abstract

Radon (^222^Rn) is a natural radioactive gas formed in rocks and soil by the decay of its parent nuclide (238-Uranium). The rate at which radon migrates to the surface, be it along faults or directly emanated from shallow soil, represents the Geogenic Radon Potential (GRP) of an area. Considering that the GRP is often linked to indoor radon risk levels, we have conducted multi-disciplinary research to: (i) define local GRPs and investigate their relationship with associated indoor Rn levels; (ii) evaluate inhaled radiation dosages and the associated risk to the inhabitants; and (iii) define radon priority areas (RPAs) as required by the Directive 2013/59/Euratom. In the framework of the EU-funded LIFE-Respire project, a large amount of data (radionuclide content, soil gas samples, terrestrial gamma, indoor radon) was collected from three municipalities located in different volcanic districts of the Lazio region (central Italy) that are characterised by low to high GRP. Results highlight the positive correlation between the radionuclide content of the outcropping rocks, the soil Rn concentrations and the presence of high indoor Rn values in areas with medium to high GRP. Data confirm that the Cimini–Vicani area has inhalation dosages that are higher than the reference value of 10 mSv/y.

## 1. Introduction

Naturally occurring background radiation is the main source of exposure for most people. Much of it comes from primordial radionuclides in the Earth’s crust, such as ^40^K, ^238^U and ^232^Th, and their associated decay products [1]. The average global dosage of natural and man-made radiation, including that from the alpha decay of radon and its progeny, is about 3 mSv. Background natural radiation is believed to account for about 80% of this total, although actual values are highly site-specific. For example, high natural background levels are found in Ramsar in Iran, Guarapari in Brazil, Karunagappalli in India [2], Arkaroola in Australia and Yangjiang in China [3]. Ramsar, a northern coastal city in Iran, has areas with some of the highest levels of natural radiation measured in populated areas (up to 260 mSv/y) [4].

Radon is a major source of ionizing radiation exposure for the general population and is known to be a risk factor for the onset of lung cancer [5,6]. Radon tends to accumulate in buildings where it can reach significant activity concentrations. The main sources of indoor radon are the soil and underlying geological units (geogenic radon) and the building materials [1]. Although the former tends to be the dominant source, the local use of building stone that is rich in radon precursors (e.g., volcanic tuff, pozzolan) means that the latter is significant in some areas of the world [7,8,9]. Relationships between indoor radon and quantities related to geogenic Rn, such as the Rn potential or uranium concentration in the ground (so-called radon predictors), need to be established because only indoor Rn is directly linked to the safety directive reference values.

The EU 2014 Basic Safety Standards describes Radon-Prone Areas (RPAs) as geographic areas or administrative regions where surveys indicate that the percentage of dwellings expected to exceed national reference levels is significantly higher than in other parts of the country [6]. However, indoor radon measurements can be biased by unconstrained, highly variable “external” factors (such as meteorological conditions, building materials, floor levels, habits of the inhabitants, etc.). These factors can provide only a partial knowledge of the risk associated with the presence of radon in confined environments. The correct strategy to realistically describe radon behaviour involves collecting information on the radon source (e.g., radionuclide content of soil/rock) and the radon migration processes that regulate its movement in the subsoil and its resulting indoor accumulation [10,11]. Although geological parameters may show a certain spatial variability at the local level (less than indoor radon, in any case), at a regional scale they show a more marked spatial stationarity (in the geostatistical sense) and are, therefore, robust predictors of the spatial behaviour of radon in nature. The identification and geostatistical processing of the geological features that characterise the subsoil scenario can thus provide information about the Geogenic Radon Potential (GRP) of an area, i.e., radon released by the Earth [12]. One of the first methods used to assess the Geogenic Radon Potential (GRP) of an area is based on a continuous variable, as originally proposed by Neznal et al. [13] and subsequently applied by other authors [6,12,14]. The GRP depends on the equilibrium of soil gas radon activity concentrations at a fixed depth (0.8–1 m) and the associated soil gas permeability. Neznal et al. [13] set three GRP categories: low (GRP < 10); medium (10 < GRP < 35); and high (35 < GRP). If these values are not available, then the radon potential is usually estimated using proxies.

Various European countries have tried to map indoor radon activity using different approaches, but these maps do not accurately represent radon spatial distribution due to its extreme variability and the clustering of the measurements. For these reasons, they are generally represented using point symbol maps or by aggregating the indoor mean value at the scale of a region, municipality, census tracts, etc. [15,16]. To overcome some of the limitations related to indoor radon spatial quantification, other more spatially continuous parameters have been examined. For example, soil gas measurements have been described, as well as the use of the “equivalent” uranium (eU) and average radium content in soil as proxies in the absence of soil gas data [10,11,17]. Geographical Weighted Regression [10] and Empirical Bayesian Regression Kriging [11,17] were used to estimate the GRP at the regional (Lazio region) and local scales (Euganean Hills) by using soil gas radon as the response variable and assuming that the radon risk depends primarily on the relationship with some proxy characteristics (e.g., geological, geochemical and environmental) of the study area. In this regard, the radionuclide content in the soil and soil permeability are important factors that should be included in the GRP conceptual model.

In this work, we evaluate the radon hazard of three different areas in the central Italian region of Lazio, each of which is characterised by different radon potential levels. The comparison between soil radon data (^222^Rn and ^220^Rn), terrestrial gamma dose rates, indoor radon and gamma dose rate is used to evaluate the effect of the single parameters and the interplay between them for hazard definition. The inhaled radiation dose from radon is also calculated to evaluate the risk to the inhabitants.

## 2. Geological Setting of the Study Areas

**Figure 1:**
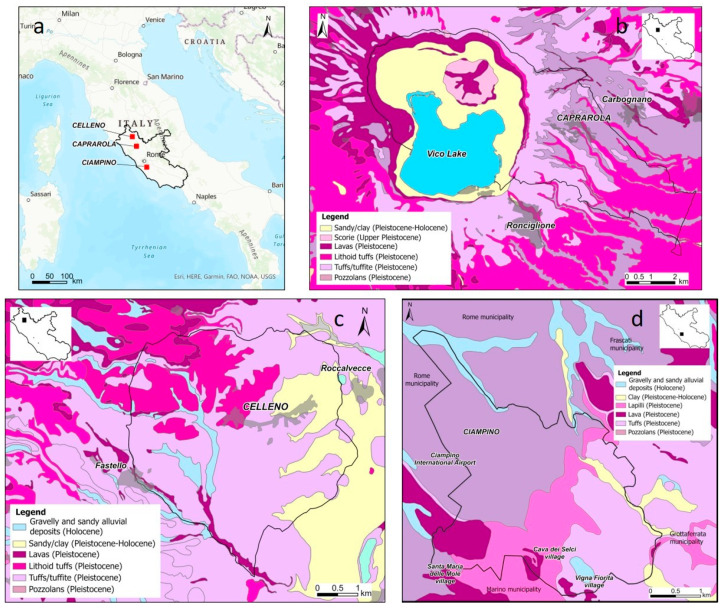
Study sites (**a**) and geological maps of the Caprarola (**b**), Celleno (**c**) and Ciampino (**d**) municipalities.

The studied municipalities of Caprarola, Celleno and Ciampino are located on the Tyrrhenian Sea margin of central Italy, within the volcanic complexes of the Cimini–Vicani Mts, the Vulsini Mts and the Alban Hills, respectively (Figure 1).

Extensional tectonic activity has controlled the evolution of the Tyrrhenian area since the Middle–Late Pliocene, resulting in dominant NW–SE and NE–SW faulting patterns [18,19] and the creation, via crustal thinning, of several NW–SE trending marine and continental sedimentary basins [20]. Associated volcanic activity during the Quaternary generated large volumes of potassic and ultrapotassic lavas and pyroclastics [21] that were deposited on horst and graben structures. An overlying sedimentary sequence comprises, from top to bottom, Miocene–Quaternary marine to continental clay and sandy-clay formations and Mesozoic carbonate formations.

The Vicano volcanic complex (0.42 − 0.09 My [22]) consists of a stratovolcano (developed on a NW–SE elongated graben at the intersection with a NE–SW fracture) with a central caldera that hosts Lake Vico. This volcanic activity alternated between explosive and effusive phases, with the production of fall deposits, lava and pyroclastic flows that were followed by circum-caldera hydromagmatic and Strombolian eruptions. The products of the Vicano complex, which consist mainly of leucitites, phono-tephrites and leucite-phonolites [23], outcrop extensively throughout the municipality of Caprarola (Figure 1b). The activity concentrations of radionuclides in lithoid ignimbrite and phreatomagmatic products from this complex are very high (up to 250 Bq/kg for ^226^Ra, 389 Bq/kg for ^232^Th and 157 Bq/kg for ^40^K; [8,9]).

The Vulsini volcanic complex (0.59 − 0.127 Ma [24,25]) was formed by deposits from the Paleobolsena, Bolsena, Latera and Montefiascone eruptive centres [26]. Activity was dominated by explosive eruptions, ranging from Strombolian and hydromagmatic events from monogenetic centres to major Plinian and pyroclastic flow-forming events associated with caldera collapse [27]. The volcanic products include a full range of potassic rock types, from K-basalts, trachybasalts, basanites and tephrites to K-foidites, phonolites and trachytes [27]. They outcrop widely on the western sector of the Celleno municipality (Figure 1c). High radionuclide concentrations are also observed in Vulsini volcanic rocks, with values up to 395 Bq/kg for ^238^U, 488 Bq/kg for ^232^Th and 2446 Bq/kg for ^40^K [28].

Most activity related to the Alban Hills volcanic complex (0.6 − 0.02 Ma [29]) took place during the Tuscolano–Artemisio (0.6 − 0.3 Ma), Campi di Annibale (0.3 − 0.2 Ma) and hydromagmatic (0.2 − 0.02 Ma) phases [29] and references therein. These phases included cycles of violent explosive eruptions with repeated caldera collapses, lava emission and the formation of eccentric craters with dominant hydromagmatic activity [29]. The products are silica poor, strongly alkaline potassic lavas, pyroclastic flows and tephra [30]. The municipality of Ciampino is situated within these products (Figure 1d). Radionuclide contents, measured in pyroclastic rocks, are up to 220 Bq/kg for ^226^Ra, 379 Bq/kg for ^232^Th and 251 Bq/kg for ^40^K [8].

## 3. Methodology

### 3.1. Site Selection

The selection of the study sites (Figure 1a) was based on a preliminary analysis of the already available Geogenic Radon Potential (GRP) map of the Lazio region [10]. The GRP is a spatially continuous quantity directly related to the local geology that, when coupled with indoor radon data, can provide a reasonable guide for identifying Radon Priority Areas (RPAs) [31,32]. The construction of the GRP of an area is based on the analysis of the spatial distribution of some proxy geological information (e.g., lithological types, U, Th and Ra content, the Rn emanation coefficient from rocks, soil/rock permeability, faults, etc.) that can be related to in situ radon production and migration processes [10,11,17,33,34,35,36,37,38,39,40]. The resulting GPR map defines the spatial distribution of radon risk from sub-surface sources; information that can then be used for land-use zoning and strategic indoor radon monitoring purposes.

In this paper, we create new Geogenic Radon Potential maps by integrating literature data [11,41] with new field data (soil gas, terrestrial gamma dose rate, radionuclide content of outcropping soil/rocks) collected during the LIFE-Respire project (LIFE 16/ENV/IT/000553) in the municipalities of Caprarola, Celleno and Ciampino. The GRP maps of the three municipalities are compared to the Terrestrial Gamma Dose Rate (TGDR) maps of the same sites and the new indoor radon measurements to evaluate the source of indoor radon and evaluate the link with the geological scenarios.

### 3.2. Field Activities and Laboratory Analyses

#### 3.2.1. Soil Gas Sampling

Soil gas sampling is a well-defined technique for the monitoring of the shallow environment [42,43]. It consists of collecting soil air samples by pounding a 6.4 mm, thick-walled, stainless steel tube into the ground to a depth of between 0.7 and 0.9 m to minimise the influence of meteorological variables [44].

Soil gas radon (^222^Rn) and thoron (^220^Rn) activities were measured with a portable RAD7 alpha detector (Durridge Company Inc., Sheffield, UK) connected to the sampling probe via a drying tube used to keep the relative humidity below 10% (e.g., [45]). The alpha detector consisted of a solid-state ion-implanted silicon semiconductor calibrated to measure in the range of 4 to 400,000 Bq/m^3^. A single measurement had an average duration of 20 min, with partial readings every 5 min.

#### 3.2.2. Gamma Dose Rate Measurements

Terrestrial (TGDR) and indoor gamma radiation (IGR) measurements were performed using two instruments. The first was a portable NaI γ-ray spectrometer, the “Exploranium GR-135 Plus Identifier”, capable of directly measuring the Ambient Dose Equivalent Rate (ADER) [11]. The second was a digital Ratemeter (model 2241-3, Ludlum Measurements, Inc., Sweetwater, TX, USA) connected to a scintillator gamma detector (model 44-11, Ludlum Measurements, Inc. Sweetwater, Texas). Both instruments were inter-calibrated by performing several measurements together. The dose rate was in the range of between 0.2 and 0.8 μSv/h, and the results were corrected according to a calibration coefficient (error was about 5% and the overall uncertainty (1 − σ) was around 8%) [46].

The outdoor measurements were made on relatively compact agricultural soils (e.g., open fields) and the surveys were conducted during dry periods with stable atmospheric pressure conditions, no or very low wind speed and avoiding the early morning hours when the accumulation of radon progeny in dew can increase the normal soil emission rate by up to 15% [47]. Two indoor measurements were performed in each monitored room at 1 m above the floor in the centre of the room and in direct contact with a wall.

#### 3.2.3. Indoor Radon Concentration Measurements

Indoor radon concentrations were measured using passive nuclear track detectors based on poly-allyl diglycol carbonate, commercially known as CR-39; this plastic material is very sensitive to the tracks of highly ionizing particles, such as alpha particles. The tracks formed by the alpha particles on the CR-39 were enlarged by a chemical etch process and then counted at the INGV Radionuclides laboratory using a RADOSYS automatic electronic microscope system. Chemical etching involves immersing the polymeric film in a 90 °C, 6.25 N NaOH bath for about 4.5 h, followed by an additional bath with distilled water and acetic acid to stabilise the traces. The density of the recorded alpha traces, analysed using an electronic optical microscope, is directly proportional to the average indoor radon concentration (expressed as Bq/m^3^) during the exposure period.

Two surveys were carried out: in the winter survey, the detector exposure time was from November 2018 to February 2019; in the summer survey, the exposure time was from July to September 2019. The summer and winter data were used to obtain the annual indoor radon concentration for each site. The surveys were conducted in buildings with different types of construction material (tuff, cement and other). Two detectors were placed in each building to ensure good data quality.

During the indoor surveys, a questionnaire was distributed to the owners that requested information about building characteristics (e.g., number of floors, building material, year of construction, foundation type, etc.) and occupant habits.

#### 3.2.4. High Resolution Gamma Spectrometry

Soil and rock samples, representative of the main lithological units present in the study areas, were analysed using a high-resolution gamma spectrometer equipped with a low-background HPGe coaxial detector (GEM-EG&G ORTEC) to determine the activity concentrations of the radionuclides ^238^U, ^226^Ra, ^232^Th and ^40^K, which were estimated from 186 keV (^235^U), 352 keV (^214^Pb), 583 keV (^208^Tl) and 1461 keV (^40^K) γ-rays, respectively, using the Capo di Bove leucitite (Alban Hills) as standard. More details on the sample preparation and isotope measurements are described in [11].

### 3.3. Assessment of Radiological Risk Parameters

The annual inhalation dose (D), expressed as mSv/y, due to exposure to the volumetric activity of indoor radon was calculated using the equation [48]:

D = IRC × F × O × DCF(1)

where IRC is the indoor radon concentration (Bq/m^3^), F is the recommended equilibrium factor value (0.4) for radon and its progeny, O is the indoor occupancy factor and DCF is the dose conversion factor (9 nSv/h per Bq/m^3^) for radon and its progeny. The occupancy factor was assumed to be 7000 h/y for homes, 2000 h/y for workplaces and 350 h/y for cellars used as storage rooms.

The outdoor/indoor Annual Effective Dose Equivalent (AEDE_out_ − AEDE_in_), expressed as mSv/y, due to the irradiation of the body by penetrating gamma rays in outdoor/indoor environments was calculated using the equation [48]:

AEDE = ADER × T × F⁄10^3^ (2)

where ADER is the mean Ambient Dose Equivalent Rate for each neighbourhood (in μSv/h), T is the number of hours in one year (8760 h/y) and F is the outdoor/indoor occupancy factor (assumed by convention to be equal to 0.2 and 0.8, respectively). AEDE_in_ was calculated using the average value of the Indoor Gamma Radiation (IGR) measured at a height of 1 m in the centre of the room; AEDE_out_ was calculated using the average value of the Terrestrial Gamma Dose Rate (TGDR) measured 1 m above the ground in an open field. The AEDE measures the risk of stochastic and deterministic effects for humans. To assess the radiological risk, the Excess Lifetime Cancer Risk (ELCR) from outdoor/indoor gamma radiation was calculated using the equation:

ELCR = AEDE × LE × RF(3)

where LE is the Life Expectancy (70 years) and RF is the Risk Factor, i.e., fatal cancer risk factor for stochastic effects (0.057 per Sievert of exposure [49]). The ELCR is defined as the probability that an individual will develop cancer due to lifetime exposure to gamma radiation.

### 3.4. Statistical Data Analysis and Mapping Techniques

#### 3.4.1. Exploratory Data Analysis (EDA)

Exploratory Data Analysis (EDA) techniques were applied to all collected data to identify interacting processes, such as anomalous values caused by particular phenomena, for example advective gas migration from a deep source, diffusive gas emissions and biological sources (e.g., [43,50,51,52]). Descriptive statistics and graphical representations were carried out to characterise the population of soil gas samples. The statistical evidence of interacting processes is represented using histograms, box plots and scatterplots coupled with the calculation of some statistical indices (e.g., minimum and maximum values, mean, geometric mean, standard deviation, coefficient of variation, etc.). Normal Probability Plots (NPP) were used to recognise anomalous samples or to define multiple populations in the data distribution [53]. Data were statistically elaborated using the Statistica software package (StatSoft, Inc., 1984–2014, Tulsa, OK, USA).

#### 3.4.2. Geostatistics and Spatial Analysis

The protocol followed in the LIFE-Respire project for the selection of the Italian study sites (Caprarola, Celleno and Ciampino) was based on the Geogenic Radon Potential (GRP) map of the Lazio region, as described in [15]. In this work, the construction of the GRP of an area is based on the analysis of the spatial distribution of radon in soil gas and some proxies, such as geological, geochemical, structural and geomorphological data from the literature and newly collected field data (e.g., soil gas, TGDR, radionuclide content of soils and rocks and permeability). These data are more robust for the construction of GRP maps because they are characterised by: (i) higher spatial autocorrelation; (ii) lower variability; and (iii) not depending on anthropogenic factors (as is the case for building and anthropogenic parameters related to indoor radon). Newly collected and available literature data were processed to create GRP maps of the three municipalities using Empirical Bayesian Regression Kriging (EBRK). EBRK is a multivariate geostatistical technique that uses simple kriging and principal component analysis in the regression model to predict the dependent variable (e.g., soil gas radon) from a series of independent variables (e.g., geological proxies). Variogram calculation is performed through subsetting and simulations to accurately predict the non-stationary variables. For specific information see [11,17,54]. The maps were constructed using a 100 × 100 m grid size, which is the average nearest neighbour distance between the closest two measurement sites. The spatial analysis and geostatistics were performed using ArcGis Pro 2.8 software (ESRI Inc. Redlands, CA, USA). See [55,56,57,58,59] for detailed information regarding the geostatistical analysis and mapping techniques used.

## 4. Results

### 4.1. Outdoor Surveys

**Figure 2:**
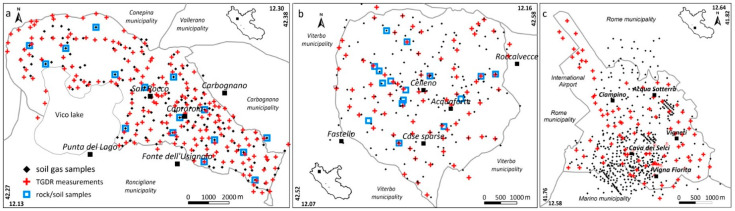
Distribution of the different samples collected in the three municipalities (Caprarola, (**a**); Celleno, (**b**); Ciampino, (**c**)): soil gas (black dots), TGDR (red crosses) and rock/soil samples for radionuclide content (blue squares).

Figure 2 shows the spatial distribution of the samples collected in the three municipalities, including 741 soil gas samples, 365 TGDR measurements and 35 rock and soil samples for radionuclide measurement in the laboratory. The adopted field strategy aimed to maintain, as much as possible, a constant sampling density for the soil gas and TGDR surveys, while the rock and soil samples are representative of the outcropping lithologies. In particular, the available dataset consists of:

- 180 soil gas samples, 187 outdoor gamma measurements and 12 rock/soil samples in the Caprarola municipality (about 57 km^2^) (new data) (Figure 2a);
- 185 soil gas samples, 82 outdoor gamma measurements and 16 rock/soil samples in the Celleno municipality (about 25 km^2^) (from [11]) (Figure 2b);
- 390 soil gas samples (270 from the literature [41,42] and 120 from this work), 96 outdoor gamma measurements (new data) and 11 rock/soil samples [8,60,61] in the Ciampino municipality (about 13 km^2^) (Figure 2c).

#### 4.1.1. Soil Gas and Terrestrial Gamma Dose Surveys

Detailed statistics of soil gas Rn data is reported in Table 1. The radon data collected in the Caprarola municipality had a very high mean value (157.9 kBq/m^3^), about two to three times greater than those calculated for the other two municipalities and three times higher than those calculated for a regional dataset covering all of Lazio (about 8000 samples) [10].

**Figure 3:**
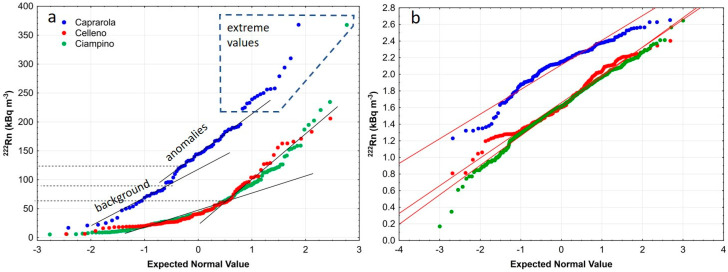
Normal Probability Plots of soil gas radon measurements (**a**) and log-transformed data (**b**).

Table 1 shows that arithmetic means are quite different from the median and geometric mean values, thus supporting the hypothesis that Rn does not follow a normal distribution. Furthermore, the large standard deviation value indicates high data variability and the skewness indicates a marked positive asymmetry for all the municipalities. These results, together with NPPs of both the raw and log-transformed data (Figure 3), indicate that the radon data has a log-normal distribution, as often reported in the literature. In addition, for Caprarola and Ciampino, the standardised skewness and kurtosis are outside the range (−2 to +2) of data expected for a normal distribution, and thus the geometric mean (GM, 130.4 kBq/m^3^) can be taken as the representative value of the soil Rn activity.

A further examination of Table 1, which includes data collected by the Fluid Geochemistry Laboratory of the Earth Sciences Department of the Sapienza University of Rome (see [41,62,63,64,65]) and by ARPA Lazio [66] throughout the Lazio region (7610 samples), shows that the data from the Caprarola site has a GM value that is six times greater than that calculated for the entire Lazio region, similar maximum values and the lowest CV value of all four datasets (indicating a more homogeneous ^222^Rn activity compared to the other two sites).

Table 2 shows the descriptive statistics of the in situ gamma ray survey (TGDR). The radiation values range between 0.154–0.970, 0.130–0.417 and 0.105–0.236 μSv/h for the Caprarola, Celleno and Ciampino municipalities, respectively. The distribution of the gamma values is essentially normal, with a slight positive skewness, similar mean, median and geometric mean values and standardised skewness and kurtosis within the range of −2 to +2, as expected for a normal distribution. However, the Shapiro–Wilk test (*p* < 0.05) suggests a non-normal data distribution (see also the highest St.Dev. and CV) for the Caprarola municipality, as commonly occurs in areas with high radiometric anomalies (e.g., [67]). The gamma dosages vary between bedrock types in all three municipalities, with volcanic lithologies having higher values than sedimentary lithologies due to their greater radionuclide content. This is in good accordance with the results of the activity concentrations of natural radionuclides and the radon distribution in soil gas.

**Figure 4:**
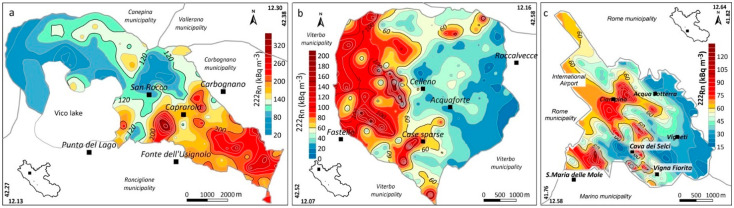
^222^Rn in soil gas maps of the Caprarola (**a**), Celleno (**b**) and Ciampino (**c**) municipalities.

**Figure 5:**
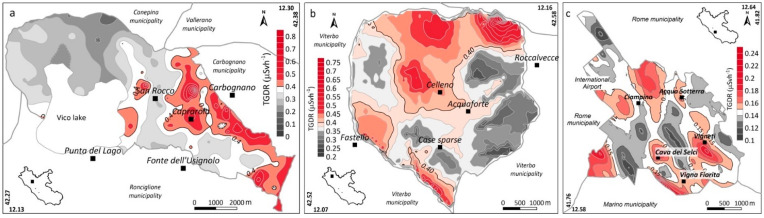
Terrestrial gamma dose rate (TGDR) maps of the Caprarola (**a**), Celleno (**b**) and Ciampino (**c**) municipalities.

Figure 4 shows the estimated ^222^Rn distribution maps of the three municipalities. In each map, the lowest bold contour line indicates the statistical anomaly threshold defined using the Normal Probability Plot technique of Sinclair [53]. Figure 5 shows the estimated contour maps of the TGDR values.

In the Caprarola municipality, the highest Rn activities mainly occur in the south-eastern sector of the investigated area (where most urban centres are located) (Figure 4a). This pattern generally agrees with the distribution of the highest TGDR values, which are linked to the outcropping lithoid volcanics having high U and Ra content. It is important to note that these rocks are used for the construction of most of the buildings in the historic centre of the Caprarola village (Figure 1). In the Celleno municipality, the high Rn anomalies mainly occur in the rural and sparsely inhabited western sector of the municipality. This area is characterised by outcropping lavas and tuffs enriched in U and Ra. Some small, elongated soil gas radon anomalies occur in correspondence with the drainage network where, at some locations, erosion has exposed the underlying lavas. TGDR anomalies also characterise part of the central and northern sectors of the municipality, showing large anomalies in correspondence with Pleistocene tuffs. Lower TGDR values occur in the western sector and trace the distribution of the outcropping marine and continental deposits. In the Ciampino municipality, the estimated Rn distribution shows fault-linked anomalies elongated according to the known NW–SE trend of the Ciampino structural high [41]. In contrast, the TGDR map shows spot anomalies that do not match the Rn anomalies, suggesting that the TGDR values are caused by the radionuclide content of the outcropping volcanic rocks while the Rn anomalies are linked to the migration of this gas along more permeable routes (e.g., faults).

#### 4.1.2. Radionuclide Content of Soil/Rock Samples

In the Caprarola municipality, the ^238^U, ^226^Ra, ^232^Th and ^40^K contents range from 85 to 369 Bq/kg, from 83 to 318 Bq/kg, from 146 to 481 Bq/kg and from 317 to 1236 Bq/kg, respectively (Table 3). High radionuclide concentrations occur in tuffs and phreatomagmatic facies; similar data have been reported in the literature for igneous rocks [48].

In the Celleno municipality, radionuclide activity concentrations reported in the literature [16] range from 42 to 281 Bq/kg, from 47 to 295 Bq/kg, from 80 to 365 Bq/kg and from 299 to 2480 Bq/kg for ^238^U, ^226^Ra, ^232^Th and ^40^K, respectively. High radionuclides concentrations occur in lava and tuff samples and the values are generally lower than those measured at Caprarola.

The content of the radiogenic elements in the Ciampino municipality was also collected from the available literature [8,60,61] and used to characterise the outcropping lithologies. The ^226^Ra, ^232^Th and ^40^K contents range from 97 to 228 Bq/kg, from 195 to 470 Bq/kg and from 85 to 264 Bq/kg, respectively; the lower values were measured in “Peperino” pyroclastic flow samples while higher values occurred in samples from “Lapilli” (fragments of pyroclastic rocks). In general, the radionuclide activity concentrations are lower than those measured at Caprarola and Celleno municipalities.

The radionuclide activity concentrations of Holocene alluvial deposits in the Caprarola and Celleno municipalities are comparable to the values of the volcanic units because they are the by-products of the weathering and erosion of these primary units.

The spatial distribution of radionuclides in the three municipalities is in good accordance with the Rn and TGDR anomalies highlighted by the maps of Figure 4 and Figure 5, respectively.

#### 4.1.3. Geogenic Radon Potential Maps

**Figure 6:**
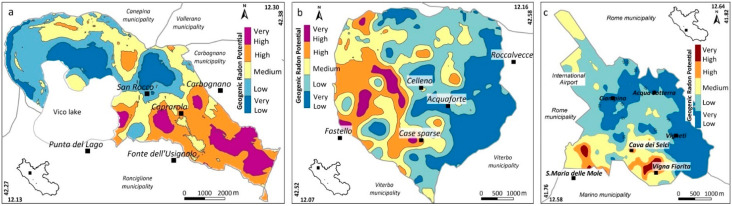
Geogenic Radon Potential (GRP) maps of the Caprarola (**a**), Celleno (**b**) and Ciampino (**c**) municipalities.

Geogenic Radon Potential maps of the three municipalities were constructed using an EBRK geostatistical model based on geological, geochemical, structural and geomorphological data collected from the literature, as well as from new field measurements conducted during this study (e.g., soil gas, TGDR, radionuclide content of soils and rocks). All data were processed using Empirical Bayesian Kriging Regression (EBKR) [11,17]. The results are shown in Figure 6. Since Rn activity in soil gas is the EBKR response variable, the GRP is expressed in kBq/m^3^. Note that the colour scale classes in Figure 6 are set using the “Jenks Natural Breaks” classification method, whereby boundaries are created in a way that groups similar values together and maximises the differences between classes (Table 4).

### 4.2. Indoor Surveys

**Figure 7:**
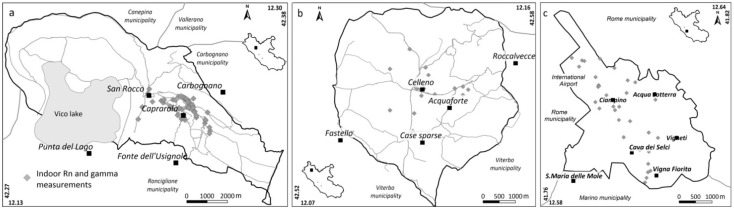
Distribution of indoor Rn and indoor gamma radiation measurements in the Caprarola (**a**), Celleno (**b**) and Ciampino municipalities (**c**).

Figure 7 shows the spatial distribution of the indoor measurements in the three municipalities. Although every effort was made to distribute the measurements homogeneously, a large proportion are constrained to the main inhabited areas. In total, we collected 466 Indoor Radon Concentration (IRC) samples and 517 Indoor Gamma Radiation (IGR) measurements.

**Figure 8:**
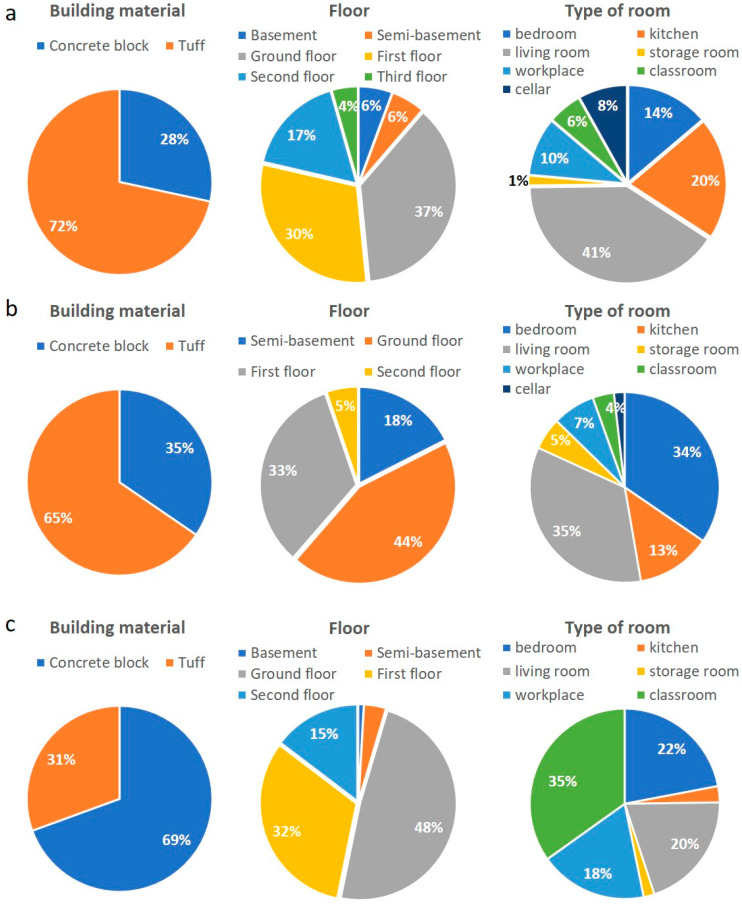
Pie charts of building materials, floor and intended use of the room in the Caprarola (**a**), Celleno (**b**) and Ciampino (**c**) municipalities.

The pie charts in Figure 8 report the building materials, floors and the intended use of the room where the IRC and IGR measurements were performed in the three municipalities. Volcanic tuff is the most used building material in Caprarola (72%) and Celleno (65%), while in Ciampino the dominant material is concrete blocks (69%) followed by lesser tuff (31%). Among the monitored levels, the ground floor prevails in all three municipalities. In Caprarola and Ciampino, the first and second floors represent 47% of the total, while in Celleno, in addition to the first floor, the semi-basement is relatively common (18%). Regarding the use of the monitored room, the living room, bedroom and kitchen prevail for Caprarola and Celleno. In Ciampino, the survey focused primarily on public buildings and thus, the classroom is the most representative (35%) followed by the bedroom and workplace.

Information about the building material, floor and room usage was used to interpret the indoor radon concentration data. Only the data from the basements and ground floors were used to investigate the correlation between IRC and GRP.

#### 4.2.1. Indoor Gamma Radiation Measurements

Table 5 shows the statistics of the indoor gamma radiation measurements carried out in the centre of the room and on a main wall of the room for each municipality. The IGR close to the wall is representative of the building material used, whereas the IGR at the centre of the room is more representative of the real gamma radiation to which people are exposed and, therefore, was chosen for statistical data processing.

The IGR values range between 0.11–1.42, 0.13–0.56 and 0.06–0.38 μSv/h for Caprarola, Celleno and Ciampino municipalities, respectively. The IGR values measured at the centre of the room are on average lower than those measured near the walls, as expected. Their statistical distribution is essentially normal, with similar mean and geometric mean values and a slight positive skewness due to the presence of a few outliers. The IGR values close to the wall range between 0.10–1.64, 0.14–0.75 and 0.06–0.42 μSv/h for Caprarola, Celleno and Ciampino municipalities, respectively. In this case, the arithmetic and geometric means and medians are also quite similar and a slight positive skewness is evident, especially in the Caprarola data. Higher IGR mean, standard deviation and coefficient of variation values for the wall measurements are linked to the building materials used (e.g., lava, tuffs and concrete), which is generally more homogeneous in the Caprarola municipality.

**Figure 9:**
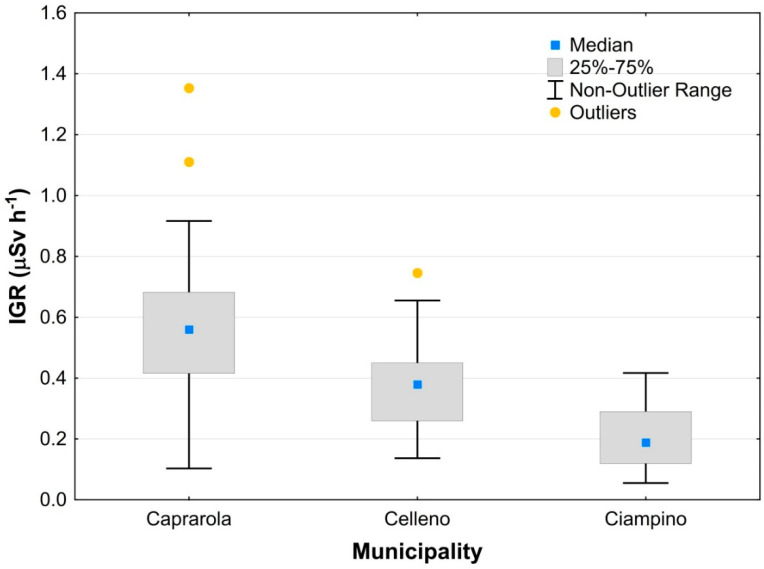
Box plot of the indoor gamma radiation data, measured at the centre of the room, in the Caprarola, Celleno and Ciampino municipalities.

Considering the IGR data relative to the building materials, buildings made of concrete blocks have average values (0.34, 0.23 and 0.12 μSv/h, for Caprarola, Celleno and Ciampino municipalities, respectively) that are lower than those measured in buildings made of tuff (0.45, 0.36 and 0.24 μSv/h). The IGR values in concrete block buildings are similar to the natural background due to the outcropping geological formations and represented by the TGDR (0.365, 0.229 and 0.148 μSv/h for Caprarola, Celleno and Ciampino municipalities, respectively; Table 2). These data suggest that the walls are the main source of Rn in the buildings made of tuff. The gamma radiation measured at Ciampino is much lower than at the other two municipalities for both measurement locations (Table 5). Among the possible explanations for this difference is the frequent use of tuff as a building material in Caprarola and Celleno, compared to Ciampino, as well as a lower background value. Overall, the indoor gamma radiation values are the highest in Caprarola, intermediate in Celleno and lowest in Ciampino (Figure 9).

#### 4.2.2. Indoor Radon Concentration Measurements

Table 6 summarises the main statistical parameters of the annual and seasonal (summer and winter) indoor radon measurements.

The IRC measurements in the Caprarola municipality were performed in 127 private and public buildings. The lowest IRC values were measured in the summer months, with a mean of 455 Bq/m^3^ and a median of 221 Bq/m^3^. The values measured in the winter period had a mean of 1091 Bq/m^3^, a median of 808 Bq/m^3^ and a maximum value of 6507 Bq/m^3^, which is twenty times higher than the EU recommended value of 300 Bq/m^3^. The mean and median values of the annual data were 701 and 470 Bq/m^3^, respectively. These IRC values are the highest measured among the three municipalities.

The IRC measurements in the Celleno municipality were performed in 44 private and public buildings. The collected data exhibited higher values in the winter, with a mean of 527 Bq/m^3^ and a median of 298 Bq/m^3^, and lower values in the summer, with a mean of 285 Bq/m^3^ and a median of 129 Bq/m^3^. The highest value of 3909 Bq/m^3^ was recorded in the summer period in a cellar carved directly into a tuff deposit. The annual mean and median values were equal to 378 and 211 Bq/m^3^, respectively. It should be noted, however, that about 40% of the measurements show average annual values above the EU recommended threshold of 300 Bq/m^3^.

The IRC measurements in the Ciampino municipality were performed in 76 private and public buildings. The lowest IRC values were observed in the summer period, with a mean of 205 Bq/m^3^ and a median of 157 Bq/m^3^. In the winter period, instead, a mean of 362 Bq/m^3^ and a median of 209 Bq/m^3^ were detected. The mean winter values were clearly higher than the mean summer values (362 and 205 Bq/m^3^, respectively), with a substantial increase in the maximum indoor values in the winter (1575 Bq/m^3^) that was twice that of the summer period (764 Bq/m^3^). The annual mean and median values (283 and 203 Bq/m^3^, respectively) are both below the EU recommended limit of 300 Bq/m^3^. It is interesting to note that the CV values for the winter measurements are very similar, suggesting a greater stability of the IRC data for the three municipalities, probably due to a more uniform behaviour of the inhabitants (such as the habit of closing the windows).

**Figure 10:**
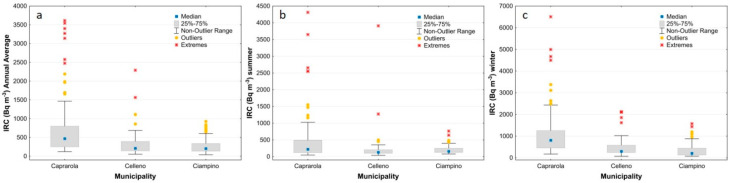
Box plots of indoor radon concentration (IRC) for annual (**a**), summer (**b**) and winter (**c**) values for the three municipalities.

**Figure 11:**
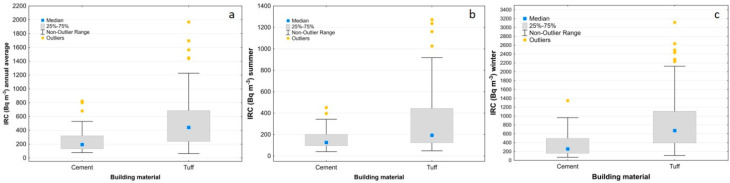
Box plots of IRC as a function of building materials, considering annual (**a**), summer (**b**) and winter (**c**) measurements.

**Figure 12:**
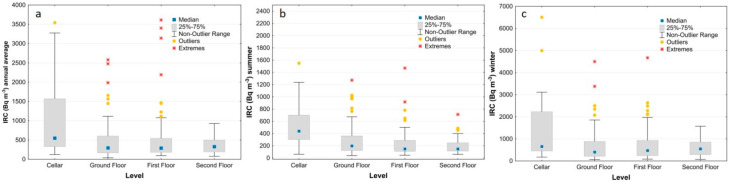
Box plots of the measured IRC for annual (**a**), summer (**b**) and winter (**c**) values carried out in all three municipalities for each monitored floor level.

The box plots of Figure 10 show that the indoor Rn values generally decrease in the summer (Figure 10b), and the differences between the three municipalities are more visible for the annual average and during the winter season (highest in Caprarola, intermediate in Celleno and lowest in Ciampino) (Figure 10a and Figure 10c, respectively). In the winter survey, about 90% of the houses in Caprarola, 51% in Celleno and 47% in Ciampino show indoor radon activity concentrations above the reference level. The box plots that show the IRC values as a function of the building material for the combined data from all three sites (Figure 11) highlight the strong effect that tuff has on indoor radon measurements, regardless of municipality or measurement period. The box plots of the IRC data as a function of floor level (Figure 12) only show a strong influence for samples collected in cellars, with these values being much higher than the European Directive’s threshold of 300 Bq/m^3^. The summer measurements (Figure 12b) show a slight decreasing trend in the IRC from ground to second floor, whereas during the winter (Figure 12c) there is little difference between the upper three levels. This latter behaviour is in part expected, probably being due to the so called “chimney effect” [68] induced by home heating during the winter. In contrast, during the summer, the distance from the possible main source of geogenic radon beneath the house becomes more important. When tuffs are present in a room’s walls, high values are also measured in the upper floors, irrespective of the season.

#### 4.2.3. Radiological Parameters

The descriptive statistics of the radiological parameters are reported in Table 7. In the Caprarola municipality, the inhalation dose values range from 0.16 to 91.05 mSv/y, with a mean value of 12.06 mSv/y. In most cases, the inhalation doses in private houses are higher than the recommended action level of 3–10 mSv/y [69], i.e., a level for which some radon mitigation is required or recommended. The dose values in working places are generally low with respect to those calculated in private houses. The mean Annual Effective Dose Equivalent (AEDE) values are 3.04 and 0.64 mSv/y for indoors and outdoors, respectively. The average Excess Lifetime Cancer Risks (ELCR) values are 11.71 and 2.55 × 10^−3^ for indoors and outdoors, respectively.

In the Celleno municipality, the inhalation dose values range from 0.35 to 39.4 mSv/y, with a mean value of 8.11 mSv/y. As for Caprarola, in most cases, the inhalation doses in private houses are higher than the recommended action level. The data from working places are limited, but values appear to be particularly low. The mean AEDE values are 2.06 and 0.40 mSv/y for indoors and outdoors, respectively. The ELCR values are 7.94 and 1.60 × 10^−3^ for indoors and outdoors, respectively.

In the Ciampino municipality, the inhalation dose values range from 0.70 to 23.35 mSv/y, with a mean value of 5.18 mSv/y. The inhalation dose values only exceed the recommended action level in a few cases, both in private and public buildings. The mean AEDE values are 1.24 and 0.26 mSv/y for indoors and outdoors, respectively. The ELCR values are 4.77 and 1.03 × 10^−3^ for indoors and outdoors, respectively.

## 5. Discussion

In this work, we started from the GRP map of the Lazio region [10] and selected three municipalities located in different GRP areas: Caprarola (high radon potential), Celleno (medium radon potential) and Ciampino (low radon potential). All collected data converge to confirm this pattern as demonstrated by the highest content of radionuclides (U, Th, Ra and K) in rocks/soil samples (Table 3), comparable TGDR (Figure 5) values for Caprarola and Celleno and the lower values of these two variables found in the Ciampino municipality. In contrast, the soil gas radon activity in the three municipalities shows concentrations of the same order of magnitude. This suggests that the hypothesis that, for the Caprarola and Celleno municipalities, the main source of radon could be due to the lithology (e.g., high content of radionuclides) while for the municipality of Ciampino, radon migrating from a deeper source, controlled by the fault system of the Ciampino structural high, could be added to the lithological contribution. This additional radon input can be linked to the presence of deeper mineral waters hosted in the carbonate Mesozoic basement as a result of secondary volcanic activity in the area and can be transported toward the surface by a continuous and/or sudden CO_2_-dominant gas release [41,64].

### 5.1. Definition of the Radon Priority Areas

**Figure 13:**
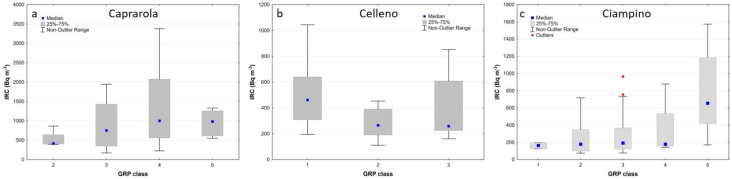
Box plots of the IRC measurements in basements and ground floors grouped in the GRP classes for each municipality. Graphs show that median values of IRC for Caprarola (**a**) and Ciampino (**c**) municipalities increase according to the GRP class; this is not evident for Celleno (**b**) municipality probably caused by the fact that the main inhabited area does not occur in higher GRP classes.

All collected geological data, including fracture and permeability measurements, were used to construct a kriging regression model to estimate the soil gas Rn concentrations (e.g., response variable) and to obtain the GRP maps at the scale of the municipality (Figure 6). The next step was to compare the GRPs of the study areas and the spatial distribution of the IRC to evaluate a possible relationship between the different geological/lithological scenarios (in terms of GRP) and the measured indoor Rn levels. To accomplish this objective, the maps of the GRP were reclassified according to the classes reported in Table 4 and the GRP classes were extracted for each IRC measuring location. The final objective of this analysis was to find a preliminary and fast procedure to determine the RPA, as required by the European Directive. As the IRC is affected by many “external” parameters (e.g., meteorological, constructive and human), only the indoor measurements collected in the basement and ground floor were used. This is because, as shown in Figure 12, they are more likely to be affected by geological phenomena and, therefore, less influenced by the “external” parameters. The graph of Figure 13 highlights the presence of a high IRC in the highest GRP classes (Figure 6), at least for the municipalities of Caprarola and Ciampino. This growing trend was not found for the municipality of Celleno. This could be due to the lower sample size and a greater sample clustering, given that the municipality of Celleno has a lower population density than the other municipalities. According to the box plot of Figure 13 and the map of Figure 6, we can suggest that the areas falling into GRP classes 4 (high) and 5 (very high) can be considered as RPAs; in particular, considering the total area of the three municipalities, about 26, 20 and 6% of Caprarola, Celleno and Ciampino, respectively.

### 5.2. Assessment of the Radiological Risk

In this paragraph, a discussion follows about the potential radiological risk due to the measured indoor and outdoor gamma radiation values and the radon activity concentrations.

Radioactivity measurements are mainly aimed to evaluate human exposure levels. A potentially significant dose can come from the short-lived progenies of radon; when radon is inhaled the progenies are deposited on the inner surfaces of the lung, with their alpha emitting decay causing cellular damage. The average inhalation dose values of all sites exceed the annual dose averages estimated for Italy and worldwide (4.5 and 3.5 mSv/y, respectively [70,71]). In most cases, the inhalation doses in private houses are higher than the recommended action level of 3–10 mSv/y [69]. The dose values in workplaces are generally low, with respect to those calculated in private houses, and below the action levels, excluding some workplaces monitored in Caprarola. In Italy, several surveys have been conducted to determine the IRC in dwellings, whereas surveys in workplaces are sparse and generally limited to particular places and/or activities, such as schools, quarries and thermal spas. The data of inhalation doses are even more rare [72,73,74], even if the protection of workers against ionizing radiation exposure was regulated by the European Directive 96/29/EURATOM, Council Directive 2013/59/EURATOM and its transposition into Decreto Legislativo 101.

**Figure 14:**
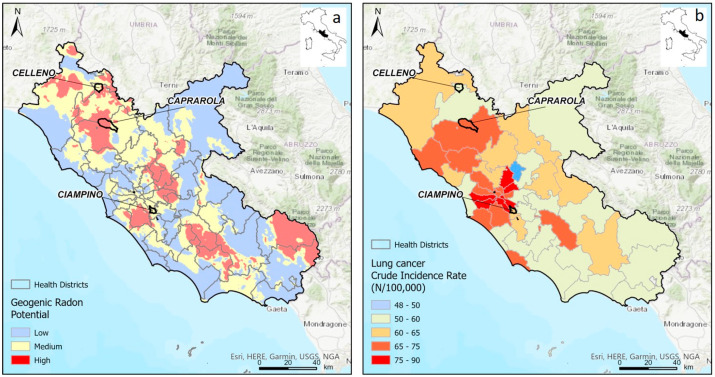
(**a**) GRP map of Lazio region, modified from original data reported in [10]; (**b**) map of Crude Incidence Rate (CIR) of tracheal, bronchus and lung cancer.

People are also significantly exposed to gamma rays, mainly from the ^226^Ra, ^232^Th and ^40^K present in terrestrial materials. The outdoor and indoor Annual Effective Dose Equivalent and Excess Lifetime Cancer Risks were calculated to evaluate the background radiation levels. The average AEDEout values are much higher than the global average of 0.106 mSv/y [48]. The average AEDEin (3.04, 2.06 and 1.24 mSv/y for Caprarola, Celleno and Ciampino, respectively) are about twenty times higher than the average outdoor values in all sites (0.64, 0.40 and 0.26 mSv/y for Caprarola, Celleno and Ciampino, respectively). The recommended total AEDE (indoors + outdoors) value should be less than 1 mSv/y [48]; this limit is largely exceeded in all sites. Similarly, the average ELCR values for all municipalities (2.55, 1.60 and 1.03 × 10^−3^ for Caprarola, Celleno and Ciampino, respectively) are higher than the world average ELCRout for outdoor exposure of 0.29 × 10^−3^ [48] and the world average ELCRtot (i.e., ELCRin + ELCRout) of 1.45 × 10^−3^ [75]. Long-term exposure to radiation is assumed to increase the risk of developing cancer. The American National Cancer Institute calculated that there is a 33% chance that a person will develop some type of cancer at some stage of their life [76]; the ELCR is the additional risk that someone might develop cancer if that person is exposed to cancer-causing materials for long periods. Therefore, according to our data, the area that is most prone to excessive lifetime cancer risk is the municipality of Caprarola, followed by Celleno and Ciampino. Considering the data on morbidity available for the Lazio region (https://www.opensalutelazio.it/salute/accessed on: 27 October 2021), the Crude Incidence Rate (CIR, i.e., the number of cases of cancer over the total population, without considering age or other factors, expressed as a rate per 100,000 persons per year) might confirm this trend (Table 8). Figure 14 shows the map of CIR of tracheal, bronchus and lung cancer in the Health Districts of the region for the period 2010–2017. The incidence of these types of cancer is higher in the Health District to which Caprarola belongs and lower in that of Celleno. In the Health District to which Ciampino belongs, the incidence is slightly higher than that of Celleno, possibly due to the high urbanization of this area (Ciampino is close to Rome). Furthermore, a comparison between the regional GRP map of the Lazio region (Figure 14a) and the CIR distribution in the health districts (Figure 14b) highlights that the higher CIR zones generally occur in correspondence with the high GRP zones.

## 6. Conclusions

The collected data confirm that the Geogenic Radon Potential is strictly linked to the geological setting of an area in terms of the radon source (e.g., radionuclide content of rock and soil), radon migration pathways (faults and fractures) and soil gas radon exhalation mechanism to the atmosphere and the indoor environment.

Multivariate spatial regression models (EBRKs) can be a robust solution to evaluate the GRP of an area by using soil gas radon as a response variable and a number of geological/geochemical proxy variables.

At the scale of the municipality, there is a good correspondence between the highest GRP areas and the highest indoor radon concentrations and gamma dose values, thus confirming that geology affects at least the lowest levels (e.g., ground and basements) of a building.

In several cases, the radiation dose received by people due to indoor radon is higher than the level for which some mitigation actions are required or recommended.

For the purposes of a preliminary assessment of the radiological risk, the Lazio GRP map shows a good correspondence with the distribution of lung cancer cases, as shown by the incidence rate map (out of 100,000) of the cases found in the Lazio region.

These areas can be considered as the Radon Priority Areas (defined in the European Directive), where municipal administrations can carry out monitoring activities at a detailed scale and adopt ad hoc remediation systems as needed.

## Figures and Tables

**Table 1:** Detailed statistics of radon data (kBq/m^3^) from the Caprarola, Celleno and Ciampino municipalities and throughout the Lazio Region. Mean (Confidence Interval 95%); Median; GM, geometric mean; Min, minimum value; Max, maximum value; St.Dev., standard deviation; G.Std.Dev., geometric standard deviation; Sk, skewness; CV, coefficient of variation; Std.Sk, standardised skewness; Std.Kur, standardised kurtosis. (Data were recalculated from [11] *, [41] **, from [10] ***, and new indices are introduced).

Site	N	Mean (CI ± 95%)	GM	Median	Min	Max	St.Dev.	G.Std.Dev.	Sk	CV	Std.Sk	Std.Kur
Caprarola	180	157.9 (142.9–168.4)	130.4	142.0	17.0	865.0	98.9	1.9	2.4	0.6	−4.9	2.3
Celleno *	185	60.0 (53.3–67.4)	45.1	40.2	6.4	253.0	48.7	2.1	1.5	0.8	1.1	−1.5
Ciampino **	120	56.2 (51.7–60.3)	41.3	41.3	1.5	444.0	48.0	2.2	2.7	0.8	−3.2	2.5
Lazio Region ***	7610	38.6 (37.4–39.8)	19.5	21.5	0.37	828.0	54.4	3.7	4.7	1.4		

**Table 2:** Detailed statistics of terrestrial gamma dose rate (μSv/h) at Caprarola, Celleno and Ciampino municipalities. Mean (Confidence Interval 95%); Median; GM, geometric mean; Min, minimum value; Max, maximum value; St.Dev., standard deviation; G.Std.Dev., geometric standard deviation; Sk, skewness; CV, coefficient of variation; Std.Sk, standardised skewness; Std.Kur, standardised kurtosis (* Data were recalculated from [11] and new indices are introduced).

Site	N	Mean (CI ± 95%)	GM	Median	Min	Max	St.Dev.	G.Std.Dev.	Sk	CV	Std.Sk	Std.Kur
Caprarola	187	0.36 (0.35–0.38)	0.35	0.34	0.15	0.97	0.11	1.34	1.36	0.31	0.18	0.35
Celleno *	82	0.23 (0.22–0.24)	0.22	0.22	0.13	0.42	0.05	1.22	0.96	0.20	0.27	0.54
Ciampino	96	0.15 (0.14–0.15)	0.15	0.15	0.10	0.24	0.02	1.15	0.97	0.14	0.25	0.49

**Table 3:** Activity concentrations of natural radionuclides (^238^U, ^226^Ra, ^232^Th, ^40^K, in Bq/kg) for the different lithologies of Caprarola, Celleno and Ciampino. * Data from [11]; ** data from [8,60,61]. When not specified, the associated uncertainties, expressed as 1σ, are of the order of 2–3% [8].

	Caprarola				Celleno *				Ciampino **			
Lithology	^238^U Bq/kg	^226^Ra Bq/kg	^232^Th Bq/kg	^40^K Bq/kg	^238^U Bq/kg	^226^Ra Bq/kg	^232^Th Bq/kg	^40^K Bq/kg	^238^U Bq/kg	^226^Ra Bq/kg	^232^Th Bq/kg	^40^K Bq/kg
Alluvial deposits (Holocene)	253 ± 5	102 ± 2	222 ± 1	642 ± 10	127 ± 2	112 ± 1	141 ± 1	457 ± 8	71 ± 3	67 ± 1	205 ± 4	916 ± 13
	144 ± 4	169 ± 2	241 ± 1	1019 ± 15	250 ± 5	261 ± 4	256 ± 2	1046 ± 13				
	139 ± 4	98 ± 2	156 ± 1	317 ± 5	80 ± 2	81 ± 1	192 ± 1	629 ± 9				
Sandy-clay (Pleistocene-Holocene)	231 ± 5	193 ± 4	356 ± 2	1236 ± 19	42 ± 1	47 ± 1	80 ± 1	572 ± 8				
	85 ± 2	83 ± 1	146 ± 1	653 ± 10	43 ± 1	46 ± 1	78 ± 1	605 ± 9				
	183 ± 4	171 ± 2	247 ± 1	921 ± 14								
Lava (Pleistocene)					196 ± 4	181 ± 2	183 ± 1	1671 ± 13				
					107 ± 2	119 ± 2	335 ± 2	1504 ± 23				
Tuff and tuffite (Pleistocene)	201 ± 4	194 ± 4	371 ± 2	774 ± 12	89 ± 2	74 ± 1	211 ± 2	1460 ± 15		158	398	187
	185 ± 4	157 ± 2	358 ± 2	766 ± 11	135 ± 4	127 ± 1	247 ± 2	798 ± 10		150	470	181
	171 ± 4	133 ± 2	238 ± 2	658 ± 10	92 ± 2	87 ± 1	249 ± 2	815 ± 12				
	129 ± 4	103 ± 2	206 ± 1	482 ± 7	281 ± 5	295 ± 4	282 ± 2	916 ± 14				
	140 ± 4	147 ± 2	299 ± 2	710 ± 11								
	102 ± 2	103 ± 2	252 ± 2	562 ± 8								
Lithoid tuff (Pleistocene)	145 ± 4	166 ± 2	218 ± 1	722 ± 11	119 ± 2	84 ± 1	182 ± 1	403 ± 8	227 ± 4	66 ± 1	204 ± 4	600 ± 13
	369 ± 6	318 ± 4	481 ± 3	1231 ± 18	78 ± 2	60 ± 1	170 ± 1	665 ± 10	45 ± 3	43 ± 41	79 ± 2	471 ± 10
	147 ± 4	145 ± 2	195 ± 1	619 ± 9	118 ± 2	124 ± 2	254 ± 2	681 ± 10	55 ± 3	58 ± 1	108 ± 2	557 ± 12
	272 ± 5	185 ± 4	304 ± 2	604 ± 9	98 ± 2	108 ± 2	216 ± 1	299 ± 4		97	195	128
					94 ± 2	89 ± 1	226 ± 1	826 ± 12				
					173 ± 4	165 ± 2	293 ± 2	1313 ± 20				
					186 ± 4	207 ± 3	365 ± 2	2480 ± 37				
					120 ± 2	121 ± 2	320 ± 2	1452 ± 22				
					53 ± 1	56 ± 1	162 ± 1	725 ± 11				
Lapilli (Pleistocene)										220	379	251
										228	376	264
Pozzolana (Pleistocene)									132 ± 3	111 ± 2	213 ± 4	1484 ± 8
										142	341	85

**Table 4:** Classification of the Geogenic Radon Potential maps. The “Jenks Natural Breaks” method was used to determine the boundaries between the soil gas Rn activity classes.

Municipality	Very Low	Low	Medium	High	Very High
Caprarola	0–70	70–110	110–160	160–220	220–360
Celleno	0–40	40–70	70–100	100–150	150–270
Ciampino	0–20	10–55	55–70	70–100	100–230

**Table 5:** Detailed statistics of indoor gamma radiation (μSv/h) at the centre of the room and close to the wall, measured in the municipalities of Caprarola, Celleno and Ciampino. N, number of samples; Mean (Confidence Interval 95%); GM, geometric mean; Median; LQ, lower quartile; UQ, upper quartile; St.Dev., standard deviation; CV, coefficient of variation; Sk, skewness.

Indoor Gamma Radiation (μSv/h)	Municipality	N	Mean (CI ± 95%)	GM	Median	Min	Max	LQ	UQ	St.Dev.	CV	Sk
Room centre	Caprarola	142	0.43 (0.40–0.46)	0.40	0.40	0.11	1.42	0.30	0.54	0.18	42.27	1.52
	Celleno	43	0.29 (0.26–0.33)	0.27	0.29	0.13	0.57	0.19	0.36	0.11	37.47	0.63
	Ciampino	74	0.18 (0.16–0.19)	0.16	0.17	0.06	0.38	0.11	0.22	0.07	41.26	0.42
	All data	259	0.34 (0.31–0.36)	0.29	0.31	0.06	1.42	0.21	0.43	0.19	55.23	1.38
Wall	Caprarola	141	0.57 (0.53–0.61)	0.53	0.56	0.10	1.64	0.42	0.68	0.22	38.49	1.23
	Celleno	43	0.37 (0.33–0.42)	0.34	0.38	0.14	0.75	0.26	0.45	0.15	40.61	0.41
	Ciampino	74	0.20 (0.18–0.23)	0.18	0.19	0.06	0.42	0.12	0.29	0.10	49.02	0.30
	All data	258	0.43 (0.40–0.46)	0.36	0.40	0.06	1.64	0.26	0.58	0.24	56.19	0.95

**Table 6:** Detailed statistics of indoor Rn (Bq/m^3^) (annual, summer and winter) measured in the municipalities of Caprarola, Celleno and Ciampino. N, number of samples; Mean (Confidence Interval 95%); GM, geometric mean; Median; LQ, lower quartile; UQ, upper quartile; St.Dev., standard deviation; CV, coefficient of variation; Sk, skewness.

Indoor Radon Concentration (Bq/m^3^)	Municipality	N	Mean (CI ± 95%)	GM	Median	Min	Max	LQ	UQ	St.Dev.	CV
Annual average	Caprarola	127	701 (573–829)	491	470	123	3613	248	800	728	104
	Celleno	44	378 (247–509)	256	211	55	2291	142	392	431	114
	Ciampino	76	283 (237–329)	227	203	40	926	134	338	202	71
Summer	Caprarola	118	455 (335–576)	259	221	49	4313	118	490	661	145
	Celleno	41	285 (92–479)	160	129	41	3909	100	206	614	215
	Ciampino	76	205 (177–233)	181	157	79	764	126	248	121	59
Winter	Caprarola	114	1091 (900–1283)	801	808	174	6507	455	1256	1032	95
	Celleno	41	527 (362–692)	373	298	70	2126	242	584	522	99
	Ciampino	76	362 (287–437)	259	209	74	1575	128	447	329	91

**Table 7:** Statistics of radiological parameters in the municipalities of Caprarola, Celleno and Ciampino. Inhalation dose (D) calculated from IRC using Equation (1); AEDE, Annual Effective Dose Equivalent, calculated from indoor and outdoor gamma radiation using Equation (2); ELCR, Excess Lifetime Cancer Risks calculated using Equation (3) * [48].

Municipality	Inhalation Dose (D) (mSv/y) (Min–Max)	AEDE_in_ (mSv/y) (Min–Max)	AEDE_out_ (mSv/y) (Min–Max)	ELCR_in_ (×10^−3^) (Min–Max)	ELCR_out_ (×10^−3^) (Min–Max)
Caprarola	12.06 (0.16–91.05)	3.04 (0.74–9.92)	0.64 (0.27–1.70)	11.71 (2.87–38.19)	2.55 (1.08–6.80)
Celleno	8.11 (0.35–39.43)	2.06 (0.92–4.03)	0.40 (0.23–0.73)	7.94 (3.55–15.50)	1.60 (0.91–2.91)
Ciampino	5.18 (0.70–23.35)	1.24 (0.44–2.68)	0.26 (0.18–0.41)	4.77 (1.70–10.33)	1.03 (0.73–1.65)
World average *	3.50	0.45	0.11	1.16	0.29

**Table 8:** Crude Incidence Rate (CIR), expressed as a rate per 100,000 persons and calculated for the years 2010–2017, considering both all types of cancer and tracheal, bronchus and lung cancer, in the Health Districts of the Lazio region (data from https://www.opensalutelazio.it/salute/accessed on: 27 October 2021).

Health District	Cancer Type	Crude Incidence Rate (CIR)
ASL Viterbo Distretto C (Caprarola)	All cancer types	643.3
	Tracheal, bronchus and lung cancer	68.4
ASL Viterbo Distretto B (Celleno)	All cancer types	633.0
	Tracheal, bronchus and lung cancer	58.8
ASL Roma 6, Distretto H3 (Ciampino)	All cancer types	630.9
	Tracheal, bronchus and lung cancer	62.1

## Data Availability

Data available on request due to restrictions, e.g., privacy. The data presented in this study are available on request from the corresponding author.
